# Nephroprotective Effect of Diosmin against Cisplatin-Induced Kidney Damage by Modulating IL-1β, IL-6, TNFα and Renal Oxidative Damage

**DOI:** 10.3390/molecules28031302

**Published:** 2023-01-30

**Authors:** Tarique Anwer, Saeed Alshahrani, Ahmad M. H. Somaili, Abdullah H. Khubrani, Rayan A. Ahmed, Abdulmajeed M. Jali, Ayed Alshamrani, Hina Rashid, Yousra Nomeir, Mohammad Khalid, Mohammad Firoz Alam

**Affiliations:** 1 Department of Pharmacology & Toxicology, College of Pharmacy, Jazan University, Jazan 45142, Saudi Arabia; 2 Department of Pharmacognosy, College of Pharmacy, Prince Sattam Bin Abdulaziz University, Alkharj 11942, Saudi Arabia

**Keywords:** nephrotoxicity, cisplatin, diosmin, lipid peroxidation, inflammatory cytokines

## Abstract

Cisplatin (CP) is a platinum compound of the alkylating agent class that is used for the treatment of various types of cancer. However, CP treatments in cancer patients are accountable for nephrotoxicity, as it is a major adverse effect. Hence, this research study was proposed to investigate the nephroprotective effect of diosmin, a flavonoid glycoside of hesperidin derivatives against cisplatin-induced kidney damage. Wistar rats received a single intraperitoneal (i.p) injection of CP (7.5 mg/kg, i.p) to induce nephrotoxicity. The administration of CP significantly (*p* < 0.001) increased the markers of kidney function test (creatinine, blood urea nitrogen, and uric acid) and demonstrated histopathological changes in the kidney of the CP-treated nephrotoxic group. In addition, the CP-treated nephrotoxic group demonstrated a significant (*p* < 0.001) increase in lipid peroxidation (LPO) levels and depleted activities of reduced glutathione (GSH), glutathione peroxidase (GPx), glutathione reductase (GR), superoxide dismutase (SOD) and catalase (CAT).However, diosmin (100 and 200 mg/kg) treatments significantly reduced the elevated levels of kidney function test parameters and restored structural changes in the kidney (*p* < 0.001). The administration of diosmin (100 and 200 mg/kg) significantly (*p* < 0.001) reduced LPO levels, increased GSH content and showed improvements in the activities of GPx, GR, SOD and CAT. The markers of inflammatory cytokines such as IL-1β, IL-6 and TNFα significantly (*p* < 0.001) increased in the CP-treated nephrotoxic group, whereas diosmin (100 and 200 mg/kg) treatments significantly (*p* < 0.001) reduced the elevated levels of these cytokines. The findings of this research demonstrate the nephroprotective effect of diosmin against CP-induced kidney damage. Therefore, we conclude that diosmin may be used as a supplement in the management of nephrotoxicity associated with CP treatments in cancer patients.

## 1. Introduction

Chemotherapy is one of the major treatment approaches for malignant solid tumors. Cisplatin (CP) is one of the most important anti-cancer drugs that is widely used in combination therapy regimens for the treatment of various malignant diseases such as head and neck, testicular, ovarian, cervical, breast, esophageal, stomach, bladder, small cell and non-small cell lung cancer [1,2]. The undesirable side effect due to CP increases morbidity and reduces the quality of life. Unfortunately, the clinical use of CP is mainly compromised due to its serious adverse effects that include nephrotoxicity, myelosuppression, severe nausea and vomiting, hearing loss and neurotoxicity [3,4]. The nephrotoxicity caused by CP in cancer patients is due to the presence of organic cation transporter-2 on the cell membrane of renal proximal tubular (RPT) cells, which actively accumulate CP and its metabolites [5].

CP-induced nephrotoxicity involves multiple pathways, such as the generation of intracellular reactive oxygen species (ROS) that triggers the cellular oxidative damage of kidney, inflammatory responses and apoptosis, which eventually contribute to renal tubular cell death and kidney dysfunction [6,7]. The important mediators of renal tubular cells damages include ROS-induced free radicals (hydroxyl radicals, hydrogen peroxide and superoxide anions). Previous studies reported that the massive production of ROS after CP treatments by renal tubular cells is possibly due to the disruption of the mitochondrial respiratory chain, the activation of NADPH oxidase and the formation of microsomes via the cytochrome P450 system [8,9]. However, other mechanisms also contribute to the pathogenesis of CP-induced nephrotoxicity, which include apparent inflammatory responses and the activation of mitogen activated protein kinase (MAPK). A recent study also reported that CP-induced nephrotoxicity is associated with the activation of the MAPK pathway that eventually plays an imperative role in the regulation of proliferation, differentiation, and apoptosis [10]. The inflammatory responses after CP treatments are a result of the activation of NF-kB, which further promotes the transcription of TNFα and other inflammatory cytokines such as IL-2, IL-6 and IL-1β [11].Hence, there is an unmet need to find safe and effective drugs that can ameliorate the serious adverse effects associated with the clinical uses of CP.

**Figure 1:**
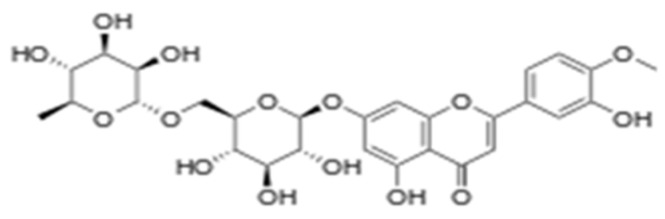
Chemical structure of diosmin.

Diosmin is an important flavonoid glycoside of hesperidin derivatives, and it is present in the peels of citrus fruit (chemical structure is depicted as Figure 1). It introduces important pharmacological activities, such as anti-oxidant, anti-inflammatory, anti-hyperglycemic, anti-mutagenic, anti-rheumatic, anti-allergic activities, etc. [12]. A recent study reported on the protective effect of diosmin against cadmium-induced liver damage [13]. In addition, Ahmed et al. [14] demonstrated that diosmin has nephroprotective potential against alloxan-induced diabetic nephropathy. Therefore, the present study was designed to evaluate the nephroprotective effect of diosmin against cisplatin-induced kidney damage by modulating IL-1β, IL-6, TNFα and renal oxidative damage in rats. 

## 2. Results

### 2.1. Diosmin and Markers of Kidney Function Test

The CP group exhibited a significant increase (*p* < 0.001) in the levels of kidney function parameters such as creatinine, BUN and uric acid levels compared to the normal control, as shown in Table 1. The administration of diosmin (100 and 200 mg/kg) demonstrated a significant decrease in the levels of kidney function parameters compared to the CP group (*p* < 0.001). 

### 2.2. Diosmin and Histological Examination of Kidney

**Figure 2:**
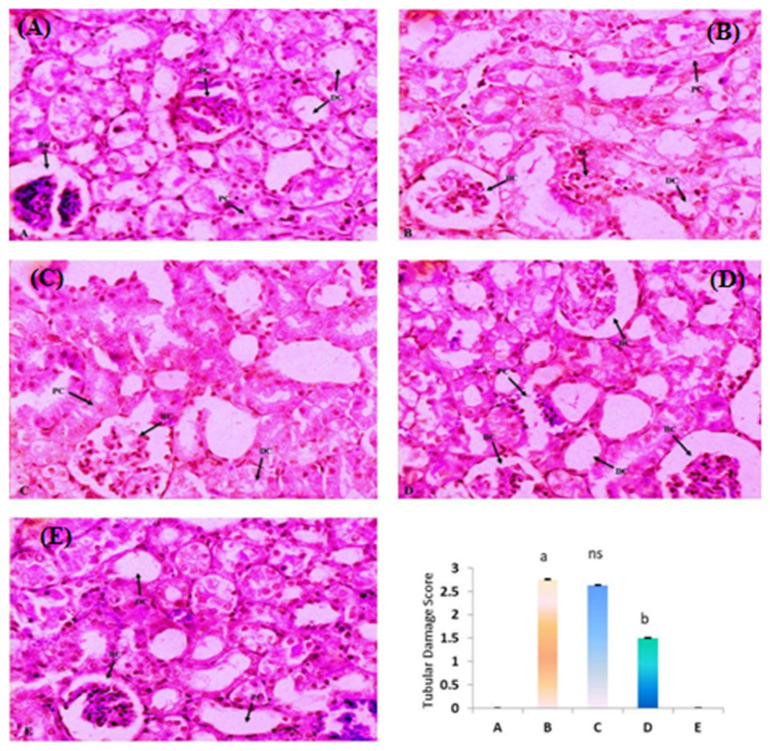
Kidney histology of normal, CP- and diosmin-treated groups. Sections of kidney tissue: (**A**) represents normal control, showing normal glomeruli and normal renal tubules without inflammation and necrosis (Score = 0). (**B**) represents the CP-treated group, showing degenerative changes in the glomeruli and renal tubules with marked tubular vacuolization and necrosis (Score = 3). (**C**) represents diosmin (100 mg/kg) + CP-treated group, showing mild degenerative changes with slight increase in the glomeruli and renal tubules size (Score = 2). (**D**) represents diosmin (200 mg/kg) + CP-treated group, showing a marked increase in the size of glomeruli and renal tubules (Score = 1). (**E**) represents the only diosmin-treated group (200 mg/kg), showing normal glomeruli and renal tubules (Score = 0). ^a^ *p* < 0.001 vs. NC, ^b^ *p* < 0.001 vs. CP, ^ns^ *p* > 0.05 vs. CP.

A histological examination of the normal and only diosmin-treated group exhibited the normal architecture of kidney without any pathological lesions showing normal glomeruli and no inflammation with normal renal tubules (Figure 2A,E). On the other hand, the CP group showed degenerative changes in the glomerular basement membrane, leukocyte infiltration with marked tubular vacuolization and necrosis (Figure 2B). Treatments with diosmin (100 and 200 mg/kg) attenuated the CP-induced degenerative changes in the kidney (Figure 2C,D). Tubular damage was examined and the tubular epithelial damage in the renal cortex was scored: 0—normal; 4—severe; 2—mild; 1—for minor. Scoring was based on tubular dilation, lesions, degeneration and tubular cell necrosis, etc.

### 2.3. Diosmin and LPO Levels

**Figure 3:**
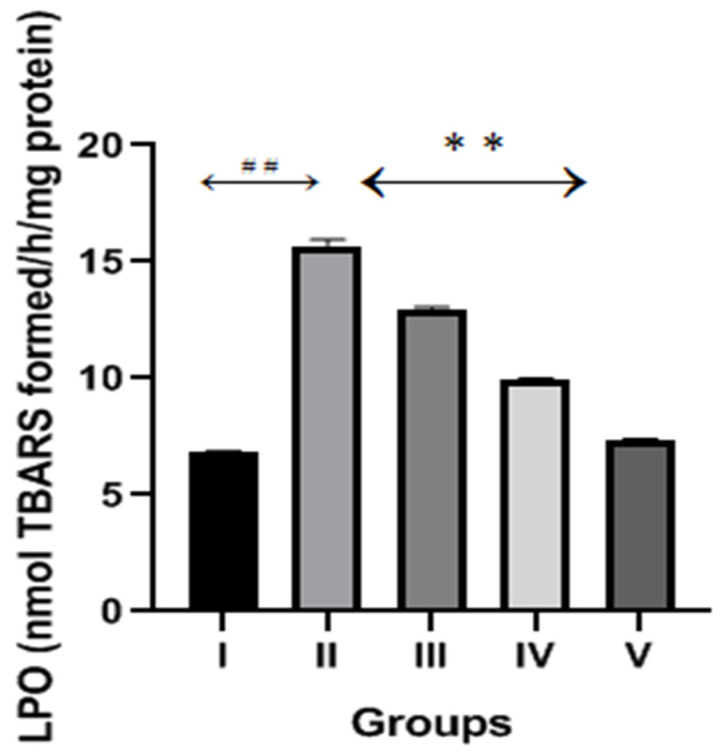
Effect of diosmin treatments on LPO levels against CP-induced nephrotoxicity in Wistar rats. Group II (nephrotoxic) compared with group I (normal)→ ^##^ *p* < 0.001. Group III and IV (treated with diosmin) compared with group II (nephrotoxic)→ ** *p* < 0.001.

Figure 3 shows the levels of LPO in the kidney tissue homogenate of normal and CP- and diosmin-treated groups. The CP group showed significant (*p* < 0.001) increase in LPO levels compared to the normal control. Diosmin (100 and 200 mg/kg) treatments significantly reduced the high levels of LPO compared to the CP group (*p* < 0.001).

### 2.4. Diosmin and GSH Contents

**Figure 4:**
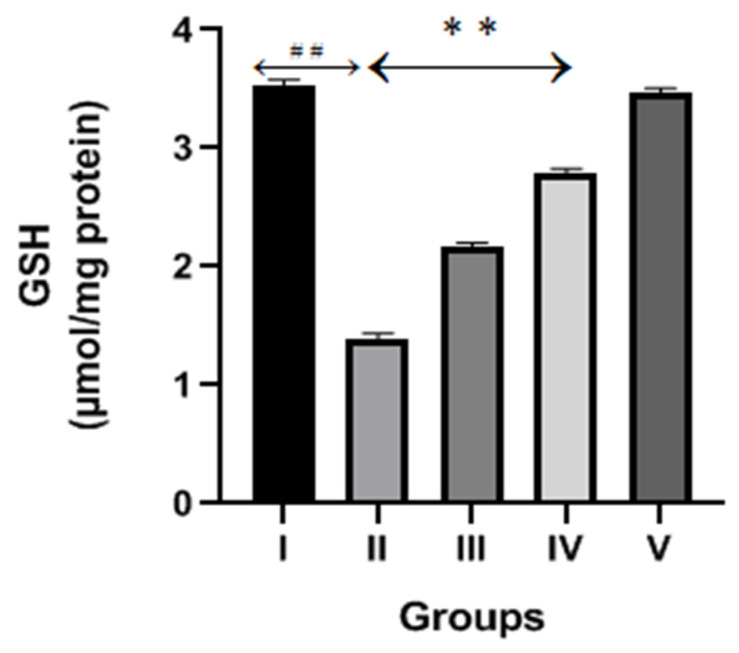
Effect of diosmin treatment on GSH contents against CP-induced nephrotoxicity in Wistar rats. Group II (nephrotoxic) compared with group I (normal)→ ^##^ *p* < 0.001. Group III and IV (treated with diosmin) compared with group II (nephrotoxic)→ ** *p* < 0.001.

The CP group demonstrated a significant (*p* < 0.001) decrease in the GSH contents compared to the normal control, as shown in Figure 4. The content of GSH significantly increased after diosmin treatments (100 and 200 mg/kg) when compared to the CP group.

### 2.5. Diosmin and Activities of Antioxidant Enzymes (AOE)

**Figure 5:**
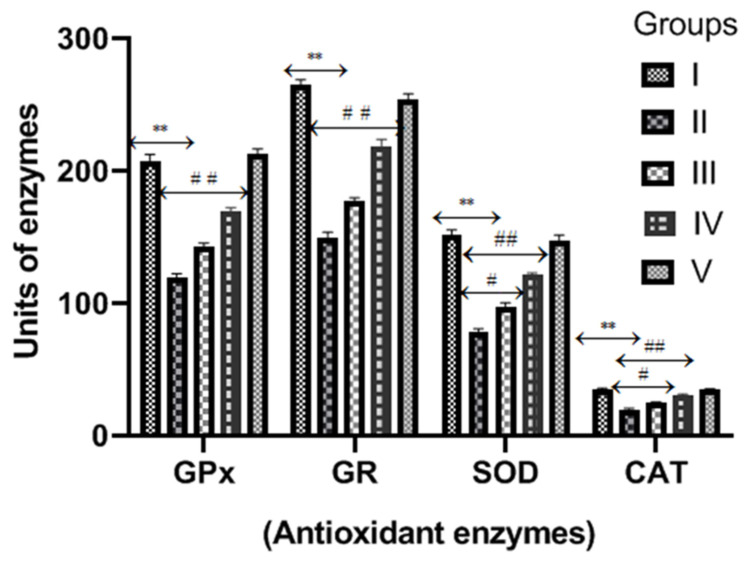
Effect of diosmin on antioxidant enzymes (GPx, GR, SOD, CAT) levels against CP-induced nephrotoxicity in Wistar rats. Group II (nephrotoxic) compared with group I (normal) → ** *p* < 0.001. Group III and IV (treated with diosmin) compared with group II (nephrotoxic) → ^#^ *p* < 0.01 → ^##^ *p* < 0.001. Units of GPx and GR: (nmol NADPH oxidized/min/mg protein); SOD: (Units/mg protein); CAT: (nmol H_2_O_2_ consumed/min/mg protein).

Figure 5 displayed antioxidant enzymes (GPx, GR, SOD and CAT) activities in the kidney tissue homogenate of the normal and CP- and diosmin-treated groups. The activities of antioxidant enzymes were significantly (*p* < 0.001) reduced in the CP group compared to the normal control. However, diosmin (100 and 200 mg/kg) administrations significantly increased the activities of GPx, GR, SOD and CAT enzymes when compared to the CP group (*p* < 0.01–*p* < 0.001).

### 2.6. Diosmin and Inflammatory Markers or Cytokines

**Figure 6:**
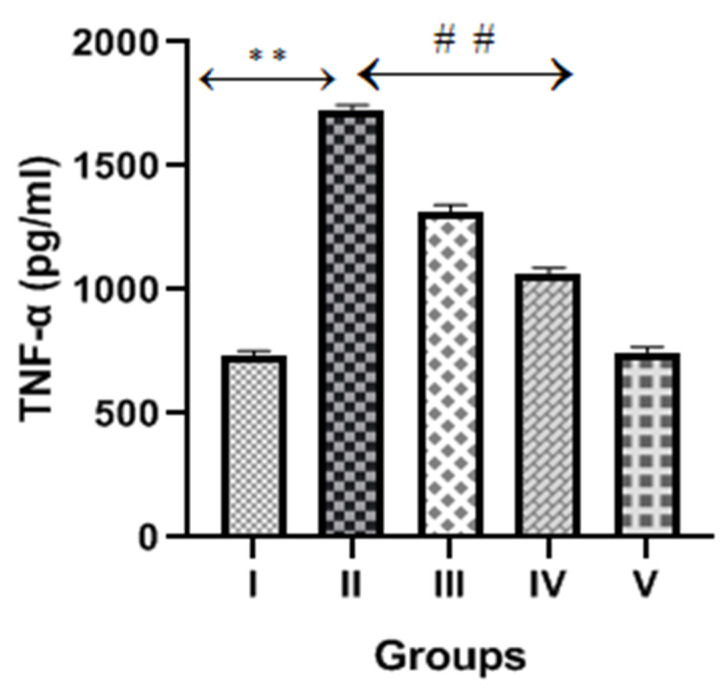
Effect of diosmin on TNFα levels against CP-induced nephrotoxicity in Wistar rats. Group II (nephrotoxic) compared with group I (normal)→ ** *p* < 0.001. Group III and IV (treated with diosmin) compared with group II (nephrotoxic)→ ^##^ *p* < 0.001.

**Figure 7:**
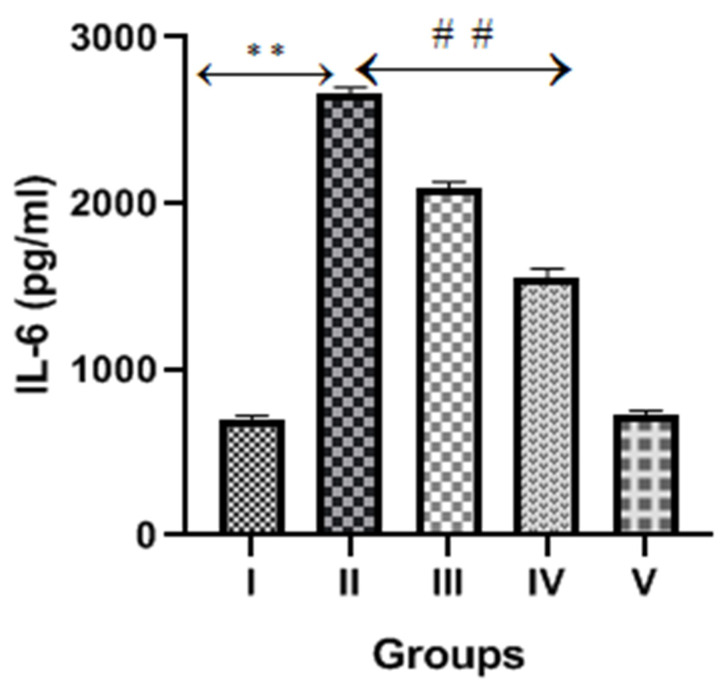
Effect of diosmin on IL-6 levels against CP-induced nephrotoxicity in Wistar rats. Group II (nephrotoxic) compared with group I (normal)→ ** *p* < 0.001. Group III and IV (treated with diosmin) compared with group II (nephrotoxic)→ ^##^ *p* < 0.001.

**Figure 8:**
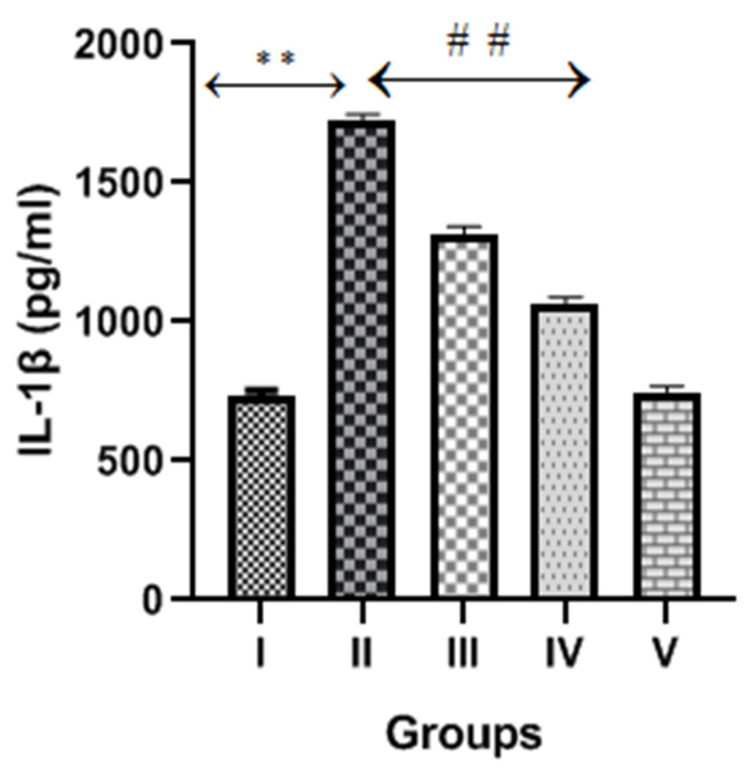
Effect of diosmin on IL-1β levels against CP-induced nephrotoxicity in Wistar rats. Group II (nephrotoxic) compared with group I (normal)→ ** *p* < 0.001. Group III and IV (treated with diosmin) compared with group II (nephrotoxic)→ ^##^ *p* < 0.001.

The CP group exhibited a significant increase (*p* < 0.001) in inflammatory cytokines markers (TNFα, IL-6 and IL-1β) compared to the normal control, as shown in Figure 6, Figure 7 and Figure 8. However, diosmin treatments (100 and 200 mg/kg) significantly reversed the elevated levels of these inflammatory cytokines compared to the CP group (*p* < 0.001). 

## 3. Materials and Methods

### 3.1. Drugs, Chemicals and Biochemicals

Diosmin and cisplatin (CP) were obtained from Sigma Aldrich Pvt Ltd., St. Louis, MO, USA. Kits for biochemicals parameters such as creatinine, BUN and uric acid were purchased from a UK-based company (Randox) via the official supplier of the country (K.S.A). ELISA kits for cytokines parameters (TNFα, IL-6 and IL-1β) were procured from a USA-based company (MyBioSource) via official supplier of the country. 

### 3.2. Animals (Wistar Rats)

Male Wistar rats within a weight range of 200–220 g were obtained from the animal house of the Medical Research Centre (MRC), Jazan University. All rats were shifted from the MRC animal house to Pharmacy College animal house for acclimatization one week prior to the experiment. The animals were kept under ideal laboratory conditions (temperature 25 ± 2 °C and humidity 45–50%) and provided with standard diets and water during experimental study. This research study was given approval by the standing committee for Scientific Research Ethics of the university (REC41/1-034).

### 3.3. Experimental Study Design

We used 40 Wistar rats for this study, and they were divided randomly into 5 groups with 8 animals in each.

Group I: normal control (NC) → received normal saline. Group II: CP-treated → received single dose of cisplatin (7.5 mg/kg, i.p) on 10th day of the treatment. Group III: Diosmin (100 mg/kg) + CP → received diosmin (100 mg/kg, p.o) daily for 14 days followed by cisplatin (7.5 mg/kg, i.p) on 10th day of the treatment. Group IV: Diosmin (200 mg/kg) + CP → received diosmin (200 mg/kg, p.o) daily for 14 days followed by cisplatin (7.5 mg/kg, i.p) on 10th day of the treatment. Group V: Only diosmin (200 mg/kg) → received higher dose of diosmin (200 mg/kg) daily for 14 days.

Blood was taken in a vacutainer tube on day 15 of the research study from overnight fasting rats. Serum was separated after the centrifugation of blood at 4000 rpm for 5 min and used for the estimation of creatinine, BUN, uric acid and cytokines such as TNFα, IL-6 and IL-1β. After blood withdrawal, all animals were sacrificed and both kidneys were excised for the assay of lipid peroxidation (LPO), reduced glutathione (GSH), glutathione peroxidase (GPx), glutathione reductase (GR), superoxide dismutase (SOD), catalase (CAT) and histopathological studies.

### 3.4. Tissue Homogenate Preparation

The homogenate of kidney was prepared in phosphate buffer (pH 7.4) and protease inhibitor (1 µg/mL). It was then centrifuged at 800× *g* for 5 min with a maintained temperature of 4 °C. The supernatant (S1) was separated from the homogenate and used for the assay of LPO and GSH. The remaining homogenate was again centrifuged at 10,500× *g* for 15 min with a maintained temperature of 4 °C to obtain a post-mitochondrial supernatant (PMS). The PMS was further used for the assay of GPx, GR, SOD and CAT.

### 3.5. Markers of Kidney Function Test

The markers of kidney function tests (Creatinine, BUN and uric acid) were estimated in serum to determine the efficiency of kidneys by using diagnostic kits from Randox, UK. The absorbance of the test sample and standard were recorded, and the values for each parameter were determined according the formula given in the kit.

### 3.6. Assay of Renal Oxidative Stress Parameters

The protein content in each sample was determined according to the method described by Lowry et al. [15] The assay for LPO was determined according to the method of Islam et al. [16].The assay for GSH was determined according to Jollow et al. [17]. The activities of GPx and GR were determined based on the methodology of Mohandas et al. [18]. The methodology of Claiborne [19] was used to determine the activity of CAT, whereas SOD activities were determined by the methodology of Marklund [20].

### 3.7. Assay of Inflammatory Markers or Cytokines

The assessments for the inflammatory markers of cytokines such as TNFα, IL-6 and IL-1β were carried out in the serum samples using the simple ELISA sandwich method according to the procedure provided in the kit. Both kidneys were homogenized, centrifuged and supernatants were prepared. The samples were added into a 96-well microplate that is pre-coated with specific antibodies of cytokines (TNFα, IL-6 and IL-1β). Then, the biotinylated detection antibody and horseradish peroxidase (HRP) conjugates were added to each well of the microplate and kept for incubation. The microplate was washed after incubation to remove free components, and the TMB substrate reagent was added. The appearance of yellow was measured by an ELISA plate reader at 450 nm wavelengths. The concentrations of these cytokines in test samples were calculated from the standard curve. 

### 3.8. Histological Examination

Kidney tissue samples were taken out immediately after the animals were sacrificed and washed with ice-cold normal saline. The tissues were then fixed in 10% formalin solution. Later the tissue was cut into appropriate size and embedded in liquid paraffin to solidify and assemble into blocks. These blocks were then used to make sections of 3–5 µm thickness with the help of microtome. The slides of these sections were prepared and stained with hematoxylin and eosin (H&E) for further histological evaluation under a microscope at 40× magnification.

### 3.9. Statistical Analysis

Analyses of data were carried out using GraphPad Prism software 8 and analyzed by ANOVA followed by Tukey’s post hoc test to ascertain statistical significances. The results were compared among each other and presented as ± standard errors mean (SEM). The minimum criterion for the results to be statistically significant was set at a value of *p* < 0.05. 

## 4. Discussion

The clinical use of CP is associated with nephrotoxicity as one of the major reported adverse effects [21]. A preliminary study was conducted in our laboratory by Tripathi et al. [11] to establish the dose of CP (7.5 mg/kg) to induce nephrotoxicity in rats. We also confirmed that CP when given as a single intraperitoneal injection ata dose of 7.5 mg/kg is capable of causing nephrotoxicity. CP and its metabolites are secreted and reabsorbed in the renal tubules, leading to their accumulation in the kidney [22]. Previous studies reported that the concentration of CP and its metabolites in proximal tubular epithelial cells (PETCs) is 4–5 times higher than in the blood [23]. The accumulation of CP and its metabolites is mainly due to the involvement of various transport-mediated uptake processes in renal PTECs. This high concentration of CP and its metabolites causes acute kidney damage possibly due to cellular oxidative damage, inflammation, vascular injury, apoptosis and proximal tubular cell damage [24]. In this context of kidney dysfunction, the process of the glomerular filtration rate (GFR) is compromised, and albumin passes into the urine, leading to its decreased levels during the clinical use of CP. At the same time, creatinine, BUN and uric acid are not filtered properly, resulting in the high levels of these markers of renal functions. Earlier clinical reports also demonstrated that the CP treatment is accountable for the decrease in GFR along with an elevation in serum creatinine, BUN and uric acid [25].

In this study, we also observed a significant increase in creatinine, BUN and uric acid levels after CP treatments, which corroborates with kidney dysfunction. The administration of diosmin at both doses exhibited a significant decrease in creatinine, BUN and uric acid. A recent study reported that pretreatments with diosmin restored the markers of renal function parameters (albumin, creatinine and BUN) in the doxorubicin-treated model of nephrotoxicity in rats [26]. Renal function parameters (albumin, creatinine and BUN) and histological changes in the kidney are considered important markers of kidney injury. The common visible structural changes in the kidney by CP, includes the degeneration of the glomerular basement membrane, tubular vacuolization, tubular epithelial necrosis and leukocyte infiltration. The administration of two doses of diosmin provides protections against CP-induced degenerative changes in the kidney. The outcome of this study clearly demonstrates the protective effect of diosmin against CP-induced nephrotoxicity.

Cellular oxidative stress has been considered as the foremost factor that also participates in the pathogenesis of CP-induced renal dysfunction [27]. CP and its metabolites are retained by the renal tubular cells during their elimination process and stimulate the generation of ROS. These ROSs, which are overproduced after the administration of the CP, impair the oxidant–antioxidant system and damage renal tubular cell structures and functions via the stimulation of lipid peroxidation and the depletion of antioxidant enzymes [28]. Several previous reports documented that LPO is an important marker of cellular oxidative stress that damages cell membrane lipids via the generation of ROS, which subsequently increases malondialdehyde (MDA) levels, a metabolic product of lipid peroxidation [29,30]. The GSH is a well-known endogenous antioxidant, which detoxifies the ROS and protects cellular systems against the deleterious effects of excessively produced lipid peroxidation. The increased production of LPO is possibly due to an excessive production of ROS and the decreased utilization of GSH [31]. Previous studies also documented the significance of oxidative stress markers and ROS in CP-induced renal cell damage and its protection with natural antioxidants [32,33]. In this research study, we also observed significantly high levels of LPO and the decreased contents of GSH in the kidney tissue homogenate of CP group. However, both doses of the diosmin treatment significantly decreased the elevated levels of LPO and increased GSH contents in a dose dependent manner. These observations suggest that diosmin protects renal oxidative damage against CP by scavenging ROS and maintaining GSH contents.

Increased renal oxidative stress and depleted antioxidant enzymes (GSH dependent enzymes, catalase and superoxide dismutase) are accountable for the generation of ROS with a significant increase in lipid peroxidation. SOD is a very important enzyme and is present in ample amounts in all cells to protect them against the renal oxidative injury resulting from ROS. It converts highly toxic superoxide anions (O_2_^.−^) to the less toxic hydrogen peroxide (H_2_O_2_), which is further detoxified by GPx and CAT [34,35]. GPx and CAT protect cells from oxidative damage by converting free H_2_O_2_ to water by utilizing proton generated from the oxidation of GSH. In order to provide more protons to protect cells from oxidative damage, GSH is oxidized to GSSG; hence, the GSH level starts depleting in cells that undergo oxidative damage. The GSH level is maintained in a cell by GR, which converts GSSG back to GSH. Depleted antioxidant enzymes reduce the clearance of H_2_O_2_ and facilitate the production of toxic hydroxyl radicals, leading to renal cell oxidative damage [36]. However, CP treatments significantly depleted the activities of antioxidant enzymes, viz., GPx, GR, SOD and CAT. Our results are also consistent with the previously published reports regarding the depleted activities of antioxidant enzymes in the CP group. In the present study, we established that both doses of diosmin augmented the activities of these antioxidant enzymes in the kidney tissue of CP-treated rats, which demonstrates the nephroprotective and antioxidant properties of diosmin.

Other than cellular oxidative stress, inflammatory pathway activations also play vital roles in the pathogenesis of CP-induced nephrotoxicity [6]. When CP and its metabolites accumulate in the kidney, it creates a microenvironment by generating ROS, which further activates transcription factor NF-kB. The activated NF-kB promotes the transcription of TNFα and other inflammatory cytokines (IL-1, IL-2 and IL-6). Several studies proposed that TNFα is the key regulator of inflammatory activities after CP exposure and is responsible for high levels of inflammatory cytokines and chemokines [37]. A previous study reported that diosmin treatments restored the expression of NF-kB and other inflammatory cytokines in the doxorubicin-treated model of nephrotoxicity [26]. In the present study, we observed significantly high levels of TNFα, IL-6 and IL-1β in CP-treated rats, suggesting the involvement of inflammatory cytokines that damage renal tubular cells. Interestingly, the levels of these inflammatory cytokines were reduced after the administration of diosmin, thus demonstrating its protective effect in CP-induced nephrotoxicity. 

## 5. Conclusions

The results obtained from this research study demonstrated the potential protective effect of diosmin against CP-induced nephrotoxicity. This is possibly induced by modulating the kidney function test parameters, IL-1β, IL-6 and TNFα; renal oxidative damage; and restoring structural changes in the kidney. Hence, we conclude that diosmin may be used as a supplement in the management of nephrotoxicity associated with CP treatments during cancer chemotherapy. Further clinical studies are required to support this research study for its clinical approval.

## Figures and Tables

**Table 1:** Effect of diosmin treatments on creatinine, BUN and uric acid against CP-induced nephrotoxicity in Wistar rats.

Groups	Treatment	Creatinine	BUN	Uric Acid
I	Normal	1.24 ± 0.03	37.30 ± 2.15	2.40 ± 0.06
II	Nephrotoxic (CP, 7.5 mg/kg, i.p)	5.01 ± 0.09 ^##^	101.50 ± 4.08 ^##^	6.07 ± 0.10 ^##^
III	Test group (received diosmin 100 mg/kg, p.o followed by cisplatin 7.5 mg/kg, i.p on 10th day of treatment.	3.95 ± 0.09 **	79.63 ± 2.47 **	4.84 ± 0.09 **
IV	Test group (received diosmin 200 mg/kg, p.o followed by cisplatin 7.5 mg/kg, i.p on 10th day of treatment.	2.67 ± 0.05 **	57.82 ± 2.24 **	3.50 ± 0.06 **
V	Only treated with diosmin (200 mg/kg, p.o)	1.18 ± 0.03	40.33 ± 1.14	2.49 ± 0.04

Group II (nephrotoxic) compared with group I (normal)→ ^##^ *p* < 0.001. Group III and IV (treated with diosmin) compared with group II (nephrotoxic)→ ** *p* < 0.001.

## Data Availability

The data are incorporated within the article and can be available on request from the corresponding author.
